# Factors associated with the mental health of early‐career dementia researchers: An international cross‐sectional survey

**DOI:** 10.1002/alz.71364

**Published:** 2026-04-16

**Authors:** Bryony Waters‐Harvey, Pascale Heins, Eithne Heffernan, Anika Wuestefeld, C. Elizabeth Shaaban, Adam Smith, Royhaan Folarin, Sara Laureen Bartels

**Affiliations:** ^1^ National Institute of Mental Health, School of Medicine University of Nottingham Nottingham UK; ^2^ Department of Psychiatry and Neuropsychology and Alzheimer Centrum Limburg, Mental Health and Neuroscience Research Institute Maastricht University Maastricht the Netherlands; ^3^ National Institute for Health and Care Research (NIHR) Nottingham Biomedical Research Centre Nottingham UK; ^4^ Hearing Sciences, Mental Health and Clinical Neurosciences, School of Medicine University of Nottingham Nottingham UK; ^5^ Clinical Memory Research Unit, Department of Clinical Sciences, Malmö Lund University Lund Sweden; ^6^ Memory Clinic Skåne University Hospital Malmö Sweden; ^7^ Department of Health Promotion and Development School of Nursing Pittsburgh Pennsylvania USA; ^8^ Department of Epidemiology, School of Public Health University of Pittsburgh Pittsburgh Pennsylvania USA; ^9^ Alzheimer's Disease Research Center University of Pittsburgh Pittsburgh Pennsylvania USA; ^10^ Institute of Neurology University College London London UK; ^11^ Division of Biomedical Sciences University of Global Health Equity (UGHE) Butaro Rwanda; ^12^ Department of Clinical Neuroscience Karolinska Institutet Stockholm Sweden; ^13^ Present address: Sheffield Institute of Translational Neuroscience, School of Medicine and Populational Health University of Sheffield Sheffield UK; ^14^ Present address: School of Sport, Exercise and Health Sciences Loughborough University Loughborough UK

**Keywords:** academic researchers, cross‐sectional survey, dementia, early career researcher, mental health, psychosocial factors, well‐being

## Abstract

**BACKGROUND:**

Early‐career researchers (ECRs) play a vital role in scientific progress; however, academic environments and personal characteristics may influence their mental health and capacity to work. This study explored factors associated with mental health among ECRs in dementia research (ECDRs).

**METHODS:**

We analyzed data from an international cross‐sectional survey of 283 ECDRs using a logistic regression to examine associations between demographic, work‐related, and psychosocial factors and self‐reported mental health conditions. Gender interactions were tested, and significant variables were included in a multivariable model.

**RESULTS:**

In this sample, imposter syndrome (odds ratio [OR] = 13.04), financial problems (OR = 3.08), being aged 25 to 34 years (OR = 3.69), and identifying as non‐heterosexual (OR = 4.10) were significantly associated with higher odds of reporting a mental health condition. No significant gender interactions were identified.

**DISCUSSION:**

Mental health among ECDRs appears particularly affected by imposter syndrome, financial strain, age, and sexual orientation. Targeted support addressing these factors may help sustain the dementia research workforce.

## Highlights

- This is the first study of mental health factors in early‐career dementia researchers.
- Imposter syndrome and financial strain strongly predict mental health difficulties.
- LGBTQAI+ researchers report greater vulnerability to mental health problems.
- Being aged 25 to 34 is linked to higher mental health risk.
- Results highlight the need for structural and targeted support strategies.

## 1 BACKGROUND

Early‐career researchers (ECRs) constitute a substantial proportion of the global academic workforce and play a critical role in advancing science.^1^ Given their central role in research, it is concerning that many ECRs experience mental health challenges. Research highlights that graduate students report higher levels of depression, anxiety, and stress than the general population.^2^, ^3^, ^4^ Moreover, ECRs are often reluctant to seek support due to concerns that disclosure could negatively affect their academic standing if they appear unable to manage academic and social pressures.^5^, ^6^

High levels of mental health challenges persist among individuals who remain in academia after completing their PhD, although prevalence is difficult to quantify due to limited research focused specifically on postdoctoral researchers. One study found that 70% of researchers and students reported stress, 34% had sought professional help for depression and anxiety, and a further 19% wished to seek help.^7^ A systematic review reported that 27% of academic staff regularly experienced burnout, with 32% to 42% of staff experiencing mental health conditions higher than in other working populations.^8^

**FIGURE 1 alz71364-fig-0001:**
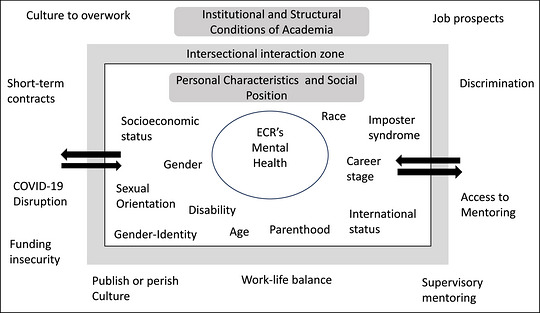
A model developed by the authors, based on the published literature, outlines personal characteristics and external factors that influence mental health in early‐career researchers (ECRs).

Multiple factors influence ECR mental health, including personal characteristics, external factors such as work conditions and academic culture, and elements that span these categories (Figure 1).

### 1.1 External factors

The nature of academia, particularly ECR roles, contributes to mental health challenges through several interconnected reasons. As universities increasingly adopt a business‐oriented model, academic success is closely tied to research output and reputation, measured by league tables (e.g., QS World University Rankings), high‐impact publications, and grant acquisition,^9^ fostering a publish‐or‐perish culture where continuous publication is essential for career advancement.^10^

In recent years, the number of PhD graduates has doubled, without a corresponding increase in academic positions,^11^, ^12^ intensifying competition for jobs, funding, resources, and mentorship.^5^ This culture, characterized by job insecurity and short‐term contracts, exacerbates stress and anxiety among ECRs.^13^, ^14^, ^15^ Financial security further compounds these pressures, as many PhD stipends, if available, fall below a living wage despite long working hours, often exceeding the standard 37‐ to 40‐h full‐time week.^16^, ^17^

Although securing research funding is central to ECRs' roles, funding opportunities are limited and often favor senior researchers, reinforcing existing hierarchies.^3^, ^4^ Grants are usually awarded based on a researcher's experience and achievements, as well as the research idea, thereby reinforcing the publish‐or‐perish culture. While ECRs frequently conduct much of the research, their contributions may be underrecognized, with credit accruing primarily to senior academics, enabling further grant success.^10^ Additionally, eligibility requirements, such as the need for long‐term contracts and the exclusion of salaries from many grants, restrict the funding available to ECRs, contributing to prolonged stress and uncertainty, as it can take many years for ECRs to gain a permanent position with regular grant acceptance.

ECRs also report difficulties maintaining work–life balance with an overwork culture, with working weeks of 60 to 80 h commonly reported, double the standard full‐time hours.^15^, ^18^ Such workloads are associated with stress, burnout, and impaired performance, creating a self‐reinforcing cycle of declining mental health.^19^, ^20^ These challenges were further exacerbated during the COVID‐19 pandemic through lab closures, contract instability, and hiring and salary freezes, which disrupted career progression and negatively affected ECRs’ mental health.^21^, ^22^, ^23^

### 1.2 Personal characteristics

While academic culture can negatively affect the mental health of all ECRs, some groups face additional challenges related to personal characteristics. Women are at higher risk of major depression, anxiety, and self‐harm, irrespective of career stage and role.^24^ Similarly, lesbian, gay, bisexual, transgender, queer/questioning, intersex, and asexual/aromantic/ally individuals (LGBTQAI+) individuals experience higher rates of anxiety, depression, suicidality, distress, and substance use than heterosexual and cisgender people.^25^

Although universities have made progress toward inclusivity, academic structures often fail to support diverse populations adequately. Women reported greater barriers in academia, including pressure to outperform male colleagues to secure or progress in their role and produce higher‐quality work, negatively affecting mental health.^26^ Career interruption due to caregiving responsibilities, including childbirth, childcare, and caring for ill relatives, further compounds these challenges.^27^ Inadequate institutional policies, including limited maternity leave, the absence of private lactation rooms, and inflexible hours, make balancing parenthood and academic responsibilities challenging, particularly when these responsibilities often coincide with critical career transitions.^28^ Navigating these constant challenges increases stress, anxiety, and burnout and may lead to women neglecting their own health to manage both demands.^29^, ^30^

Underrepresented groups, across ethnicity, disability, gender, or sexual orientation, may also face discrimination, bias, and harassment, leading to unequal access to resources, support, and opportunities and negatively affecting mental health and career progression.^27^ Despite a diverse ECR population, perceived inequality at senior levels can foster feelings of being “othered” and beliefs that senior positions are unattainable.^31^

Once again, the COVID‐19 pandemic exacerbated challenges for underrepresented groups, further worsening their mental health.^21^ For example, school and nursery closures forced families, predominantly mothers, to coordinate their research and childcare responsibilities at home, resulting in increased pressure and stress.^22^

### 1.3 ECRs in the field of dementia research and study aim

Dementia is a global health crisis, with diagnoses rapidly increasing.^32^ Advances in prevention and treatment depend on a productive academic workforce, predominantly comprising early‐career dementia researchers (ECDRs). Although the mental health of researchers has been examined in the literature, no studies – to our knowledge – have explicitly focused on ECDRs. While mental health challenges are a recognized systemic issue across the global academic workforce, researchers in medicine and health‐related disciplines, such as dementia research, often experience a higher prevalence of these conditions compared to those in other scientific field.^33^ Given the evidence that researchers in medicine and health‐related fields often experience a higher prevalence of mental health conditions than those in other disciplines,^33^ it is important to examine the factors influencing mental health in dementia research. Accordingly, this study explores personal and external factors associated with ECRD's mental health, providing a foundation for future causal association research and intervention development to support well‐being and productivity in this group.

## 2 METHODS

### 2.1 ECR definition

Despite ECRs' significant contribution to the academic workforce, there is no universally agreed‐upon definition. ECRs have been defined through self‐identification,^34^ specific roles such as postgraduate students or postdoctoral researchers,^35^ or time since PhD completion, though proposed cut‐off points vary widely.^36^, ^37^ Further disagreement exists regarding whether ECR status includes subtenure and early faculty positions, such as assistant professor and lecturer.^38^

**RESEARCH IN CONTEXT**

1. **Systematic review**: We reviewed the literature on ECRs’ mental health using established databases (e.g., PubMed). Prior work documents high levels of anxiety, depression, burnout, and financial strain among ECRs, but evidence specific to dementia researchers is scarce. Existing studies largely focus on doctoral students or academic staff broadly, with limited attention to psychosocial factors such as imposter syndrome, sexual orientation, and perceived marginalization within dementia research contexts.
2. **Interpretation**: This study extends current knowledge by presenting findings on the associations between demographic, work‐related, and psychosocial factors and mental health among ECDRs. Impostor syndrome, financial strain, being aged 25 to 34, and identifying as non‐heterosexual were strongly associated with mental health conditions, highlighting both structural and identity‐related vulnerabilities.
3. **Future directions**: Future research should use longitudinal and mixed‐methods designs with larger, more diverse samples, particularly LGBTQAI+ researchers, to clarify causal pathways and evaluate interventions addressing imposter syndrome and financial and employment security to support well‐being and workforce sustainability.

In this study, ECRs were defined as self‐identified individuals, including students, research assistants, postdoctoral fellows, and early/pre‐tenure faculty (assistant professors). Senior roles (e.g., professors) and non‐research positions were excluded.

### 2.2 Study design

The Alzheimer's Association International Society to Advance Alzheimer's Research and Treatment (ISTAART) Professional Interest Area (PIA) to Elevate Early Career Researchers (PEERs), in collaboration with University College London, conducted a cross‐sectional international online survey between September and October 2021 (during the later stages of the third COVID‐19 wave, according to the World Health Organization). The survey, delivered in English via SurveyMonkey (www.surveymonkey.com), covered several topics, including experiences, job and workplace, conference attendance, publishing, relocation, leaving academia, and the impact of COVID‐19. Ethical approval was obtained from the University College London Research Ethics Committee (21275/001).[Fig alz71364-fig-0001]

A non‐peer‐reviewed report presenting descriptive findings has been published,^39^ and subsequent peer‐reviewed papers have examined specific aspects of ECR experience using the same dataset and described the methods in greater detail.^22^, ^40^ The present study contributes new secondary analyses focusing specifically on factors associated with ECRs' mental health.

### 2.3 Participants and procedure

Individuals were eligible if they self‐identified as ECRs, currently working in any multidisciplinary field of dementia research, or had left the field within the previous 2 years. The survey could be paused and resumed later using the same web browser, and no compensation was offered. It was distributed globally via departments, institutions, networks, and charities via social media, newsletters, podcasts, blogs, and emails targeting ECDRs. At the beginning of the survey, participants reviewed the study information and data‐use statement, confirmed eligibility, and provided informed consent. Participation was voluntary and anonymous; no personal identifiers, including names and contact details, were collected. Questions were non‐mandatory, and survey branching meant that participants did not necessarily answer every item.

### 2.4 Survey data

For the present analyses, we used data from the original survey reported by Smith,^41^ including age, gender, perceived underrepresentation, ethnic minority status, job title, sexual orientation, relocation to a new country, financial problems, imposter syndrome, discrimination, and mental health. These variables were selected based on the existing literature and the researchers' conceptual model outlining personal and external factors affecting ECRs' mental health.

### 2.5 Statistical analysis

#### 2.5.1 Data preparation

**FIGURE 2:**
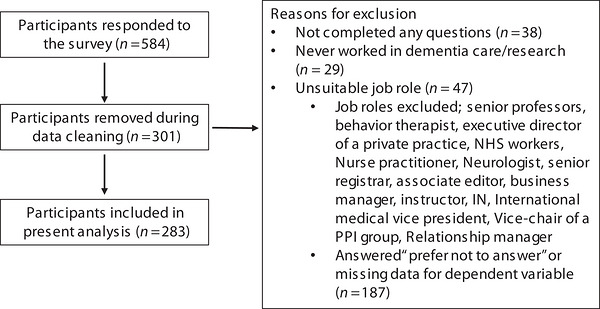
Flow diagram of excluded participants.

Although participants were asked to confirm that they met the eligibility criteria before starting the survey, a small number were excluded from the analysis because their responses indicated ineligibility (Figure 2). For example, some did not hold research roles (e.g., health professionals) or held senior roles (e.g., professors), some had not worked in dementia research, and others did not provide data for the included variables.

Seven participants identified as a gender other than man or woman, of which four had missing data on key variables. Given the small sample size and our analytical plan to examine gender interactions, it was not possible to include these participants in the regression analysis.

The survey asked about experiences of specific types of discrimination (e.g., ableism, ageism, racism, homophobia, sexism, and religious discrimination). Because the dataset within each category was small, these items were collapsed into a single discrimination variable. Similarly, the three questions asking whether participants considered themselves underrepresented where they live, at their institution, and among peers were combined into a single underrepresentation variable. Sexual orientation was collapsed from heterosexual, gay or lesbian, bisexual, questioning, and self‐described sexual orientation into two categories – heterosexual and LGBQA+ – to ensure sufficient numbers within categories for statistical analysis.

#### 2.5.2 Analyses

Descriptive statistics are presented as numbers and percentages. We examined associations between personal characteristics and work conditions and self‐reported mental health condition (yes/no) using logistic regression models. First, individual logistic regression models were run for each independent variable (age, gender, discrimination, relocation, contract length, time left of contract, imposter syndrome, financial problems, perceived underrepresentation, ethnicity, and sexual orientation). Second, for each independent variable, we ran logistic regression models that included the variable, gender, and their interaction term. Variables that were significantly associated with having a mental health condition in the individual (univariable) models were then entered into a multivariable logistic regression model. Any significant gender interactions identified in the individual models were also included in the final multivariable model to create an overall model. Analyses were conducted using SPSS version 28. All tests of significance were two‐tailed, with alpha set at 0.05.

## 3 RESULTS

### 3.1 Sample characteristics

This study included 283 participants. Of this sample, the majority were PhD students/graduate students (38.9%) or working as postdoctoral researchers (35%), aged between 25 and 34 (54.1%), women (69.6%), and heterosexual (81.6%). Further details on the sample characteristics are presented in Table 1. Participants came from 31 different countries, representing 43 nationalities. See Supplement 1 for the numbers of each nationality and country.

**TABLE 1:** Sample characteristics (*n* = 283).

Variable	Total *N* (%)	Women *N* (%)	Men *N* (%)	Other gender *N* (%)
***N***	283	197 (69.6)	83 (29.3)	3 (1.1)
**Position**				
Undergraduate	14 (4.9)	7 (3.7)	7 (8.4)	0
PhD/graduate student	110 (38.9)	84 (42.6)	26 (31.3)	0
Postdoc researcher	99 (35)	70 (35.5)	27(32.3)	2 (66.7)
Associate professor	39 (13.8)	21(10.7)	17 (20.5)	1 (33.3)
Other	21(7.4)	15 (7.6)	6 (7.2)	0
**Age (years)**				
<25	27 (9.5)	17 (8.6)	10 (12.0)	0
25 to 34	153 (54.1)	114 (57.9)	37 (44.6)	2 (66.7)
35 to 44	80 (28.3)	53 (26.9)	26 (31.3)	1(33.3)
>45	23 (8.1)	13 (6.6)	10 (12.0)	0
**Sexual orientation**				
Heterosexual	231 (81.6)	166 (84.3)	65 (78.3)	0
LGBTQAI+	52 (18.4)	31 (15.7)	18 (21.7)	3 (100)
**Racial minority**				
Yes	53 (18.7)	34 (17.3)	19 (22.9)	0
No	230 (81.3)	163 (82.7)	64 (77.1)	3 (100)
**Underrepresented**				
**Group**	133 (47.0)	104 (52.8)	27 (32.5)	2 (66.7)
Yes	135 (47.7)	86 (43.7)	48 (65.8)	1 (33.3)
No	15 (5.3)	7 (3.6)	8 (9.6)	
Missing				
**Relocation**				
Yes	142 (50.2)	102 (52.3)	38 (45.8)	2 (66.7)
No	141 (49.8)	95 (48.2)	45 (54.2)	1 (33.3)
**Dependents under 18**				
Yes	58 (20.5)	35 (17.8)	23 (27.7)	0
No	222 (78.4)	161 (81.7)	58 (69.9)	3 (100)
Missing	3 (1.1)	1 (0.5)	2 (2.4)	0
**Length of contract**				
Up to 1 year	65 (23.0)	48 (24.4)	17 (20.5)	0
2 to 3 years	112 (39.8)	82 (41.6)	29 (34.9)	1 (33.3)
4 to 5 years	64 (22.6)	44 (22.3)	20 (24.1)	0
Permanent	28 (9.9)	13 (6.6)	13 (15.7)	2 (66.7)
Student/unemployed	4 (1.4)	3 (1.5)	1 (1.2)	0
Missing	10 (3.5)	7 (3.6)	3 (3.6)	0
**Time left on contract**				
<1 year	112 (39.6)	85 (43.1)	27 (32.5)	0
1 to 3 years	107 (37.8)	78 (39.6)	28 (33.7)	1 (33.3)
3+	20 (7.1)	9 (4.6)	9 (10.8)	0
Permanent	28 (9.9)	12 (6.1)	12 (14.5)	1 (33.3)
Missing	16 (5.7)	13 (6.6)	2 (2.4)	1 (33.3)
**Happy in role**				
Happy, slightly happy	214 (75.6)	151 (76.6)	61 (73.5)	2 (66.7)
Neither happy nor sad	30 (10.6)	14 (7.1)	16 (19.3)	0
Slightly sad, sad	30 (10.6)	25 (12.7)	4 (4.8)	1 (33.3)
Missing	9 (3.2)	7 (3.6)	2 (2.4)	0
**Imposter syndrome**				
Yes	219 (77.4)	161 (81.7)	55 (66.3)	3 (100)
No	64 (22.6)	36 (18.3)	28 (33.7)	0
**Managing imposter syndrome**				
Very well, well	126 (44.5)	88 (44.7)	36 (43.4)	2 (66.7)
Neither well nor poorly	59 (20.8)	50 (25.4)	8 (9.6)	1 (33.3)
Poorly, very poorly	38 (13.4)	28 (14.2)	10 (12.0)	0
Missing	60 (21.2)	31 (15.7)	29 (34.9)	0
**Mental health**				
Yes	169 (59.7)	125 (63.5)	41 (49.4)	3 (100)
No	114 (40.3)	72 (36.5)	42 (50.6)	0
**Managing mental health**				
Very well, well	96 (33.9)	71 (36.0)	23 (27.7)	2 (66.7)
Neither well nor poorly	35 (12.4)	28 (14.2)	7 (8.4)	0
Poorly, very poorly	25 (8.8)	17 (8)	7 (8.4)	1 (33.3)
Missing	127 (44.9)	81 (41.1)	50 (60.2)	0
**Financial problems**				
Yes	104 (36.7)	62 (31.5)	41 (49.4)	2 (66.7)
No	179 (63.3)	135 (68.5)	42 (50.6)	1 (33.3)
**Experienced any form of discrimination**				
Yes	153 (54.1)	119 (60.4)	31 (37.3)	3 (100)
No	130 (45.9)	78 (39.6)	52 (62.7)	0
**Sexism**				
Yes	105 (37.1)	99 (50.3)	4 (4.8)	2 (66.7)
No	178 (62.9)	98 (49.7)	79 (95.2)	1 (33.3)
**Religious discrimination**				
Yes	14 (4.9)	6 (3.0)	7 (8.4)	1 (33.3)
No	268 (95.3)	190 (96.4)	76 (91.6)	2 (66.7)
Missing	1 (0.4)	1 (0.5)	0	0
**Racism**				
Yes	31 (11.0)	17 (8.6)	14 (16.9)	0
No	250 (88.3)	179 (90.9)	68 (81.9)	3 (100)
Missing	2 (0.7)	1 (0.5)	1 (1.2)	0
**Homophobia**				
Yes	15 (5.3)	5 (2.5)	7 (8.4)	3 (100)
No	267 (99.6)	191 (97.0)	76 (91.6)	0
Missing	1 (.4)	1 (0.5)	0	0
**Ageism**				
Yes	66 (23.3)	48 (24.4)	17 (8.6)	1 (33.3)
No	216 (76.3)	148 (75.1)	66 (79.5)	2 (66.7)
Missing	1 (.4)	1 (0.5)	0	0
**Ableism**				
Yes	13 (4.6)	9 (4.6)	3 (3.6)	1 (33.3)
No	269 (951)	187 (94.9)	80 (96.4)	2 (66.7)
Missing	1 (0.4)	1 (0.5)	0	0

*Note*: For sexual orientation, LGBTQAI+ includes lesbian, gay, bisexual, questioning, and self‐described. Transgender individuals are covered under the gender category. Data were missing for ableism, ageism, homophobia, racism, sexism, and religion; however, as these were not directly included in the analysis, the participants were retained in the final sample if they had responded yes to one of the questions.

In total, *n* = 169 participants (59.7%) reported having mental health conditions. Of those, *n* = 50 individuals reported having one condition, *n* = 58 had two conditions, *n* = 26 had three conditions, *n* = 13 had four conditions, *n* = 5 had five conditions, and *n* = 17 did not answer (Table 2).

**TABLE 2:** Type of mental health issue respondents (*n* = 169) reported experiencing.

Mental health issue	Number
Anxiety	104 (61.5%)
Depression	79 (46.7%)
ADHD	12 (7.1%)
Panic disorder	19 (11.2%)
Addiction	3 (1.8%)
Eating disorder	22 (13.0%)
Loneliness	50 (29.6%)
Mood disorder	23 (13.6%)
Other (including burnout, PTSD, OCD, sadness, stress, and sleep problems)	9 (5.3%)

*Note*: Respondents could report more than one condition.

Abbreviations: ADHD, attention‐deficit/hyperactivity disorder; OCD, obsessive‐compulsive disorder; PTSD, post‐traumatic stress disorder

Just over a third of participants (33.9%) reported managing their mental health well or very well, 12.4% reported managing it “neither well nor poorly,” and 8.8% reported managing it poorly or very poorly. Of the participants surveyed, 44.5% reported managing imposter syndrome well, while 13.5% struggled, and 20.8% managed neither well nor poorly.

### 3.2 Individual factors associated with mental health in ECDRs

**FIGURE 3 alz71364-fig-0003:**
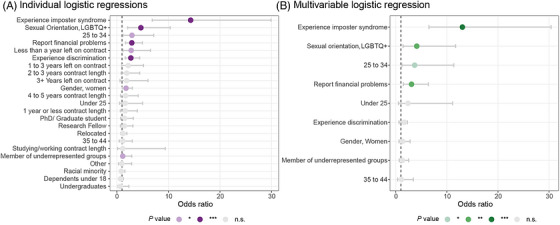
Logistic regression analyses of predictors of mental health difficulties among early career researchers (ECRs). (A) Results from individual logistic regressions examining the association between each predictor and mental health difficulties. (B) Multivariable logistic regression including all predictors entered simultaneously. Points represent odds ratios with horizontal bars indicating 95% confidence intervals. Color shading denotes significance level (*p < 0.05, **p < 0.01, ***p < 0.001, n.s. = not significant).

Logistic regression models were carried out one at a time to assess associations of each of the factors with the likelihood of reporting a mental health condition. The individual models that were statistically significant when compared to the null model were age (*X* ^2^ [3] = 13.5, *p* = 0.004), gender (*X* ^2^ [2] = 4.74, *p* = 0.03), sexual orientation (*X* ^2^ [1] = 18.25, *p* < 0.001), underrepresented (*X* ^2^ [1] = 5.16, *p* = 0.02), impostor syndrome (*X* ^2^ [1] = 68.908, *p* < 0.001), financial problems (*X* ^2^ [1] = 16.5, *p* < 0.001), and discrimination (*X* ^2^ [1] = 16.46, *p* < .001). Position, racial minority, dependents under 18, relocation, contract length, and time left in contract were not significant. See Table 3 and Figure 3A for details.

**TABLE 3:** Individual models between each factor and self‐reported mental health conditions in respondents.

Variable	*N*	Wald	Df	*p* value	Odds ratio	95% confidence interval
Position	283	3.37	4	0.497		
Undergraduates			1	0.533	0.675	0.196 to 2.322
PhD/graduate student			1	0.275	1.515	0.719 to 3.190
Research fellow			1	0.328	1.459	0.684 to 3.112
Other			1	0.985	0.985	0.341 to 2.878
Age	283	13.21	3	0.004*		
Under 25			1	0.396	1.63	0.53 to 4.98
25 to 34			1	0.018*	2.93	1.20 to 7.16
35 to 44			1	0.733	1.18	0.46 to 2.99
Gender, women	280		1	0.030*	1.78	1.06 to 2.99
Sexual orientation, LGBTQAI+	283		1	<0.001***	4.66	2.10 to 10.34
Member of an underrepresented group	268		1	0.024*	1.14	1.08 to 2.90
Experience imposter syndrome	283		1	<0.001***	14.31	6.85 to 29.91
Reported financial problems	283		1	<0.001***	2.901	1.70 to 4.94
Experience discrimination	283		1	<0.001***	2.715	1.64 to 4.43
Racial minority	230		1	0.608	0.854	0.47 to 1.56
Dependents under 18	280		1	0.254	0.712	0.40 to 1.28
Relocated	283		1	0.602	1.207	0.75 to 1.94
Contract length	273		4	0.645		
1 year or less			1	0.287	1.624	0.666 to 3.961
2 to 3 years			1	0.125	1.923	0.834 to 4.434
4 to 5 years			1	0.252	1.686	0.689 to 4.126
Studying/working			1	0.894	1.154	0.142 to 9.385
Time left on contract	267	5.683	3	0.128		
Less than a year			1	0.018*	2.782	1.188 to 6.516
1 to 3 years			1	0.067	2.213	0.945 to 5.180
3+ years			1	0.284	1.889	0.591 to 6.040

Abbreviations: Df, degrees of freedom; LGBTQAI+, lesbian, gay, bisexual, transgender, queer/questioning, intersex, and asexual/aromantic/ally individuals.

### 3.3 Gender interaction with individual factors

As previous research highlighted differences in genders, we re‐ran the individual logistic models, including the interaction between variable and gender. No variable interactions with gender were significant. See Table 4 for details.

**TABLE 4 alz71364-tbl-0004:** Gender interaction for each factor and self‐reported mental health conditions in respondents.

Variable	Df	Wald	*p* value
Position_gender	5	4.91	0.427
Age_gender	3	6.66	0.603
Underrepresented group_gender	1	0.107	0.743
Sexual orientation_gender	1	0.004	0.952
Time left on contract gender	3	4.61	0.203
Imposter syndrome_gender	1	1.83	0.179
Financial problems_gender	1	0.700	0.403
Racial minority_gender	1	0.001	0.978
Relocation_gender	1	0.59	0.442
Contract length_gender	4	1.42	0.842
Discrimination_gender	1	0.12	0.735
Dependents under 18_gender	1	0.705	0.401

Abbreviation: Df, degrees of freedom.

### 3.4 Multivariable logistic model

The final model was statistically significant compared to the null model (*X* ^2^ [7] = 104.614, *p* < 0.001), explained 44% of the variance in having mental health conditions (Nagelkerke *R* ^2^), and correctly predicted 76.6% of the cases. Having financial problems (*p* = 0.002), experiencing imposter syndrome (*p* < 0.001), identifying as non‐heterosexual (*p* = 0.009), and being aged 25 to 34 (*p* = 0.023) were significantly associated with greater odds of reporting a mental health condition, while perceptions of oneself as being underrepresented, experiencing discrimination, and gender were not significantly associated. See Table 5 and Figure 3B for details

**TABLE 5 alz71364-tbl-0005:** Multivariable model between factors and self‐reported mental health condition.

Variable	Wald	Df	*p* value	Odds ratio	95% confident interval
Age	12.80	3	0.005		
Under 25		1	0.270	2.38	0.51 to 11.11
25 to 34		1	0.023	3.69	1.20 to 11.37
35 to 44		1	0.877	1.09	0.35 to 3.43
Member of underrepresented groups		1	0.411	1.31	0.69 to 2.52
Sexual orientation, LGBTQAI+		1	0.009	4.10	1.43 to 11.73
Experience imposter syndrome		1	<0.001	13.044	6.50 to 30.39
Report financial problems		1	0.002	3.08	1.50 to 6.36
Experience discrimination		1	0.130	1.67	0.63 to 2.26
Gender, women		1	0.465	1.32	0.63 to 2.80

Abbreviations: Df, degrees of freedom, LGBTQAI+, lesbian, gay, bisexual, transgender, queer/questioning, intersex, and asexual/aromantic/ally individuals.

## 4 DISCUSSION

This study explored personal and external factors associated with mental health among ECDRs. The findings suggest that in the present sample of ECDRs, experiencing imposter syndrome, financial issues, being aged 25 to 34, and identifying as non‐heterosexual were associated with increased odds of having a mental health condition. Other factors, including racial minority status, discrimination, other age ranges, and work‐specific factors like contract length, relocation, and position, were not significantly associated in this sample. The results are similar to those of other research exploring mental health in ECRs from other fields, the self‐reported prevalence of mental health conditions was higher than reported in other studies (59.7% vs 32% to 42%)^4^, ^8^, and the impact of imposter syndrome in this sample was substantially more pronounced than in general academic populations. However, we were unable to determine whether these results are due to being a dementia researcher or another variable.

Although being a woman was associated with mental health conditions in the unadjusted analysis, this association was non‐significant in the multivariable model, suggesting mediation by other factors. Thus, the mechanisms driving the elevated mental health conditions in women appear to operate through these mediating factors rather than solely through the effect of gender itself.

Imposter syndrome, reported by 77% of participants, involves feeling inadequate or fraudulent despite evident competence and is linked to stress, anxiety, depression, burnout, and low self‐esteem.^41^, ^42^, ^43^, ^44^, ^45^, ^46^, ^47^, ^48^, ^49^ Individuals with imposter syndrome tend to attribute accomplishments to external factors, such as luck, the kindness of others, or deceit, rather than to their own abilities.^50^ The results from this study are consistent with previous research across a range of professions (e.g., academics, clinicians, health science students).^42^, ^43^, ^44^, ^49^, ^51^ The causal relationship between imposter syndrome and mental health remains unclear, warranting further research.^44^

Financial problems were another significant variable. ECRs face widespread instability, including delayed reimbursements, limited funding, and competition for grants, with marginalized groups and parents disproportionately affected.^2^, ^39^ Previous researchers highlighted support for income and funding generation as one of the top three critical needs for ECDRs.^52^ Contract length in postdoctoral research can also contribute to financial instability. While contract length was not significantly associated with mental health conditions in the current study, the variability in funding and job opportunities could contribute to career uncertainty, leading to anxiety and stress for ECRs, exacerbated by challenges in obtaining grants and a mismatch between the number of PhD graduates and available postdoctoral positions.^4^, ^53^ The COVID‐19 pandemic exacerbated financial problems, disproportionately negatively affecting ECRs both personally and professionally.^54^ Early research during the pandemic predicted significant financial losses for institutions, potentially leading to hiring freezes and layoffs, with ECRs particularly vulnerable. Our survey was conducted during the third wave of the pandemic, and thus, the impact of COVID‐19 must be considered. Many ECRs feared that the pandemic would adversely affect their career progression, thereby creating financial concerns.^53^ Lockdowns paused research activities and forced many ECRs to secure funding extensions or additional funding amid pre‐existing challenges and funding cuts.^54^, ^55^ Using the same survey data, our group previously reported that 41.8% of participants indicated that funding and job limitations negatively affected their career progression.^22^ These funding and career concerns can increase anxiety among ECRs.^54^ The predominance of fixed‐term contracts in our sample (245 of 273 participants) may explain why contract length itself was not predictive.

Being aged 25 to 34 was associated with higher odds of reporting a mental health condition, likely reflecting a period of overlapping career and personal life stressors. Individuals in this age range are typically completing a PhD or in early postdoctoral roles,^56^, ^57^ often facing short‐term contracts, job insecurity,^58^, ^59^ and life transitions such as marriage, parenthood, or homeownership.^60^ These combined pressures may contribute to the elevated prevalence of mental health conditions observed in this group.^61^ In Van der Weijden et al.^61^, postdocs reported that role uncertainty was complicated when they had children, especially when they had to relocate after their contracts ended. The current study did not ask about factors in an individual's personal life that could influence mental health conditions. Therefore, it is difficult to determine whether an increased mental health in individuals aged 25 to 34 is linked explicitly to ECRs.[Fig alz71364-fig-0003], [Table alz71364-tbl-0004], [Table alz71364-tbl-0005]

Protective factors can potentially mitigate these risks and negative impacts. Resilience can help reduce depression, anxiety, and sleep issues when individuals are faced with adversities.^62^ Social, emotional, and instrumental support from supervisors, peers, friends, or family can reduce the risk of mental health conditions, help manage challenges, and support individuals' career progression.^63^, ^64^ Participants reported relying primarily on friends and colleagues (82.6%) and mentors, advisors, and supervisors (52.2%) to manage imposter syndrome and on non‐work friends or family for mental health (70.8%) and financial concerns (60.3%).^39^ Formal institutional support was limited, with few participants finding their institution helpful in managing imposter syndrome or mental health, highlighting a clear need for structural interventions such as mentorship programs, peer networks, and accessible mental health services.

A strong sense of belonging to the research community and supervisory support that balances guidance with independence were important protective factors.^63^, ^64^ Survey results indicated that while some ECRs managed their mental health and imposter syndrome well, a substantial proportion continued to struggle, reflecting uneven coping across the cohort.

Other research into ECRs' mental health showed similar trends. A recent German survey reported high rates of anxiety and depression among postdocs, substantially higher than the German population average and an increase from their 2022 survey.^65^ The survey further highlighted financial insecurity, immigration bureaucracy, and limited career prospects as significant challenges. Our findings mirror these results, suggesting that ECDRs’ challenges reflect a broad systemic issue in academia.

### 4.1 Recommendations to address factors that influence mental health

While this study cannot determine causal relationships between factors and mental health, previous research recommended strategies and interventions to address imposter syndrome and financial issues, which could subsequently improve ECR mental health. Future research should further assess causal relationships and potential avenues for intervention.

#### 4.1.1 Imposter syndrome

Interventions can operate at individual (e.g., cognitive reframing, journaling, skill‐building, therapy), peer (workshops, discussion groups, communities of practice), and organizational levels (supervisory training, mentorship, addressing systemic biases, small grants, reducing a “failure culture”).^43^, ^44^, ^45^, ^46^, ^47^, ^48^, ^49^, ^50^, ^51^, ^52^ These approaches aim to raise awareness, normalize discussion, foster belonging and empathy, and facilitate shared learning and collaboration.^66^, ^67^ However, openly discussing imposter syndrome can pose challenges for marginalized staff and students, who may require additional support. Institutional approaches are vital as systemic factors often drive imposter syndrome.^43^, ^68^ High‐quality quantitative and qualitative research is needed to identify the most effective interventions.^66^

#### 4.1.2 Financial strain

Structural reforms are required to improve financial stability, including addressing limited funding, short‐term contracts, and precarious postdoctoral positions.^52^ Recommendations include block grants to universities to fund salaries rather than individual competition for grants, transition funding after the PhD is completed, alignment of pay with living costs, and streamlined reimbursement systems.^39^, ^52^

### 4.2 Strength and limitations

To our knowledge, this is the first study to explore self‐reported mental health conditions in ECDRs, linking multiple internal and external factors. Key strengths include detailed participant characterization, allowing assessment of independent associations across numerous factors, and the survey's global reach, with responses from 31 countries through collaborations with organizations such as ISTAART and Dementia Researcher. However, most responses came from the USA, UK, and the Netherlands, limiting generalizability to other regions. The sample was also gender‐skewed toward women. This aligns with online survey response trends^69^ and the predominance of women in care and social research fields.^39^, ^70^, ^71^ Small numbers of men and non‐binary participants limited statistical power and prevented full statistical analysis in these groups, highlighting the need for future research to recruit sufficient participants from these groups for subgroup analyses.

Mental health was self‐reported without requiring a clinical diagnosis, and timing or duration of conditions was not captured, limiting conclusions about academia's influence. Prospectively validated mental health questionnaires are recommended in addition to self‐reports. Lastly, collapsing discrimination data into a total score restricted insight into specific discriminatory experiences, underscoring the need for research examining detailed discrimination experiences and their association with mental health in ECDRs.

### 4.3 Future research

ECDRS’ mental health is complex and influenced by multiple factors. While this study identified several factors, the limited sample size and post hoc approach restricted the number of factors that could be explored. For example, future research may wish to gather data on research track versus tenure track, as they can have different expectations that could influence mental health. Further research should also validate the proposed conceptual model, notably by exploring protective factors and recruiting larger, more diverse samples, including participants from non‐Western contexts, to develop recommendations tailored to different sociocultural environments.

This study only reported associations, preventing conclusions about causality. Participants were not asked about perceived contributors to their mental health, highlighting the need for longitudinal studies examining both personal and professional factors and their interactions to clarify mechanisms and inform interventions. With only seven non‐binary participants, insight into this group was limited. However, there is evidence of high mental health vulnerability in this group.^72^, ^73^ Future researchers should explore mental health factors in non‐binary ECDRs and the adequacy of tailored support provided in academia.

## 5 CONCLUSION

Mental health conditions are more prevalent among academics, especially in ECRs, than in the general population, with the general population prevalence being 29%,^2^, ^3^, ^4^, ^5^, ^9^ potentially due to higher rates of imposter syndrome, financial problems, and discrimination, which may be due to the academic structure, such as long hours, a publish‐or‐perish culture, and competition for grants.^4^, ^13^, ^14^, ^15^, ^16^, ^17^ This prevalence may be even higher in healthcare disciplines.^74^ As dementia rates rise and a cure has yet to be discovered, it is crucial to address mental health challenges among ECDRs to ensure a robust research community that can conduct high‐quality research to prevent, diagnose, and treat dementia. This study identified imposter syndrome, financial problems, and sexual orientation as key factors associated with ECDRs′ mental health, aligning with previous research. To build on the present findings, future research should focus on longitudinal designs where causality can be determined and on increasing the external validity of study findings to diverse groups. In the future, support targeting these factors on individual, group, and organizational levels are needed to promote the mental health and well‐being of ECDRs and, thus, ensure a sustained workforce in dementia research and practice.

## CONFLICT OF INTEREST STATEMENT

EH is a member of the INTERDEM Academy Management Board. CES is a member of the ISTAART Advisory Council and Co‐chair of the Sex and Gender Special Interest Group of the Diversity and Disparities PIA in ISTAART. AW is Communication Chair of the ISTAART Neuroimaging PIA. SB is the chair of INTERDEM Academy and current (2024 to 2026) program's chair of ISTAART PEERs. The other authors have no conflicts of interest to declare. Author disclosures are available in the Supporting Information.

## CONSENT STATEMENT

All participants provided informed consent before taking part in the study.

## Supporting information



Supporting file 1: alz71364‐sup‐0001‐ICMJE.pdf

Supporting file 2: alz71364‐sup‐0002‐SuppMat.docx
