# Supplementary material for: Factors associated with the mental health of early‐career dementia researchers: An international cross‐sectional survey
Source: Alzheimers Dement. 2026 Apr 16;22(4):e71364. doi: 10.1002/alz.71364 (PMC13084526; doi:10.1002/alz.71364)
Supplement: Supplementary file 2 — Supporting file 2: alz71364‐sup‐0002‐SuppMat.docx [file ALZ-22-e71364-s001.docx]

Supplementary 1 Number of participants from each country and of each nationality represented in the study

| Nationalities | N | Countries | N |
| --- | --- | --- | --- |
| American | 48 | United States of America | 80 |
| Argentine | 4 | Argentina | 3 |
| Australian | 7 | Australia | 8 |
| Belgian | 3 | Belgium | 1 |
| Brazilian | 23 | Brazil | 20 |
| Canadian | 7 | Canada | 4 |
| Chilean | 1 | Chili | 1 |
| Chinese | 10 | China | 7 |
| Colombian | 1 | Colombia | 1 |
| Czech | 1 | Czech Republic | 1 |
| Danish | 4 | Denmark | 4 |
| Dutch | 17 | The Netherlands | 20 |
| French | 4 | France | 2 |
| German | 13 | Germany | 8 |
| Ghanaian | 1 | Ghana | 1 |
| Indian | 5 | India | 2 |
| Irish | 9 | Ireland | 3 |
| Israeli | 1 | Israel | 1 |
| Italian | 4 | Italy | 1 |
| Mexican | 4 | Mexico | 2 |
| Nigerian | 13 | Nigeria | 11 |
| Norwegian | 1 | Norway | 2 |
| Portuguese | 5 | Portugal | 1 |
| Rwandan | 1 | Rwanda | 1 |
| Salvadorean | 1 | El Salvador | 1 |
| Spanish | 5 | Spain | 3 |
| Swedish | 1 | Sweden | 10 |
| Taiwanese | 1 | Taiwan | 1 |
| Ugandan | 1 | Uganda | 1 |
| English | 47 | United Kingdom | 78 |
| Scottish | 9 | Cyprus | 1 |
| Welsh | 1 |  |  |
| South Korean | 3 |  |  |
| Cuban | 1 |  |  |
| Swiss | 1 |  |  |
| Peruvian | 1 |  |  |
| New Zealander | 1 |  |  |
| Puerto Rican | 1 |  |  |
| Guyanese | 1 |  |  |
| Greek | 1 |  |  |
| Indonesian | 1 |  |  |
| Iraqi | 1 |  |  |
